# Complete genome sequence of *Mesorhizobium* sp. strain ORM16 from *Ononis repens* root nodules reveals a distinct lineage

**DOI:** 10.1128/mra.00745-25

**Published:** 2025-09-26

**Authors:** Soufiane Alami, Mouad Lamrabet, Kaoutar Kaddouri, Zohra Chaddad, Mustapha Missbah El Idrissi

**Affiliations:** 1 Center of Plant and Microbial Biotechnology, Biodiversity and Environment, Faculty of Sciences, Mohammed V University in Rabat, Rabat, Morocco; University of Wisconsin-Madison, Madison, Wisconsin, USA

**Keywords:** *Mesorhizobium *sp., *Ononis repens*, WGS, GridION, nodules

## Abstract

*Mesorhizobium* sp. strain ORM16 was isolated from root nodules of *Ononis repens,* which grows wild in the Maamora cork oak forest (Rabat, Morocco). The complete genomic characterization enhances our understanding of the genetic basis of plant–microbe interactions in Mediterranean forest environments and contributes to the molecular taxonomy of rhizobial microsymbionts.

## ANNOUNCEMENT

The complete genome of *Mesorhizobium* sp. strain ORM16 is reported herein. This phylogenetically distinct strain, identified through molecular studies, was isolated from *Ononis repens* (common restharrow) root nodules in Morocco’s Maamora cork oak forest (34.027416°N, 6.691070°W), located near the capital city of Rabat (1).

Root nodules collected via the trapping method were subjected to surface sterilization using 0.1% mercuric chloride (HgCl₂) for one minute, washed, crushed, and streaked onto YEMA medium, with incubation maintained at 28°C (2). Bacterial isolates were purified by repeated single-colony selection and were re-streaked on solid YEM medium until pure cultures were obtained and then preserved at 4°C. The bacterial strain was identified prior to genome sequencing by 16S rRNA and multilocus sequence analysis (MLSA) analyses with phenotypic tests (1).

Genomic DNA extraction was performed from liquid cultures grown in TY medium (3) utilizing a PureLink Genomic DNA MiniKit (Invitrogen), with DNA concentration determined via NanoDrop ND2000/2000c spectrophotometry. DNA libraries were prepared using the Rapid Barcoding Kit designed for twelve samples (SQK-RBK004 × 12, Oxford Nanopore Technologies, Oxford, UK) (4). Sequencing was conducted using multiplexed samples (four samples total) on a FLO-MIN106D flow cell (R9.4.1) for 72 hours using the GridION X5 device (Oxford Nanopore Technologies, ONT). Real-time base calling was executed through Dorado software (v.7.3.11) integrated with MinKNOW (v.5.9.18), with read quality assessment performed using LongQC (v.1.2.1) (5).

The sequencing run produced a total of 62,904 reads with an N50 length of 9,919 base pairs (detailed in Table 1). Adapter removal was performed using Porechop software (v0.2.4) (6) followed by read filtering with NanoFilt (v.2.8.0) to eliminate sequences shorter than 100 base pairs (7), resulting in 58,611 high-quality reads. Genome assembly was accomplished using the Flye *de novo* assembler (v.2.9.3-b1797) (8), which generated two distinct contigs. Assembly refinement involved four iterative rounds of error correction using Racon (v.1.5.0), followed by a final polishing step with Medaka (v.2.0.1) (9). Assembly quality metrics were calculated using QUAST (v.5.2.0) (10). Default parameters were used except where otherwise noted. The final assembly comprised a circular chromosome of 6,633,211 bp with 63% GC content and a circular plasmid of 119,439 bp with a GC content of 60.5%. The circular design was proven effective through assembly. The annotation was performed using the NCBI Prokaryotic Genome Annotation Pipeline (PGAP v.6.8) (11).

**TABLE 1:** Genome sequencing project information for *Mesorhizobium* sp. strain ORM16

Strain characteristics	*Mesorhizobium* sp. ORM16
Sampling site	Cork Oak Maamora forest, Rabat, Morocco
Host plant	*Ononis repens*
No. of reads obtained	62,904
No. of reads (post-cleaning)	58,611
No. of contigs	2
Genome length (bp)	6,752,650
Chromosome length (bp)	6,633,211
Plasmid length (bp)	119,439
Coverage (×)	50
N_50_ (bp)	9,919
GC content (%)	63.0
BUSCO score (%)	97.6
ANI value to closest species %	87.23
Best match type-strain	*Mesorhizobium opportunistum* WSM2075^T^
CDSs (Coding DNA sequences)	6,585
No. of rRNAs	6
No. of tRNAs	51
No. of tmRNAs	1
BioSample ID	SAMN44467667
BioProject ID	PRJNA1178300
NCBI RefSeq assembly	GCF_044998965.1
GenBank ID	NZ_CP173356.1
Accession number	CP173356.1
Sequence Read Archive (SRA)	SRR31173793

**Fig 1:**
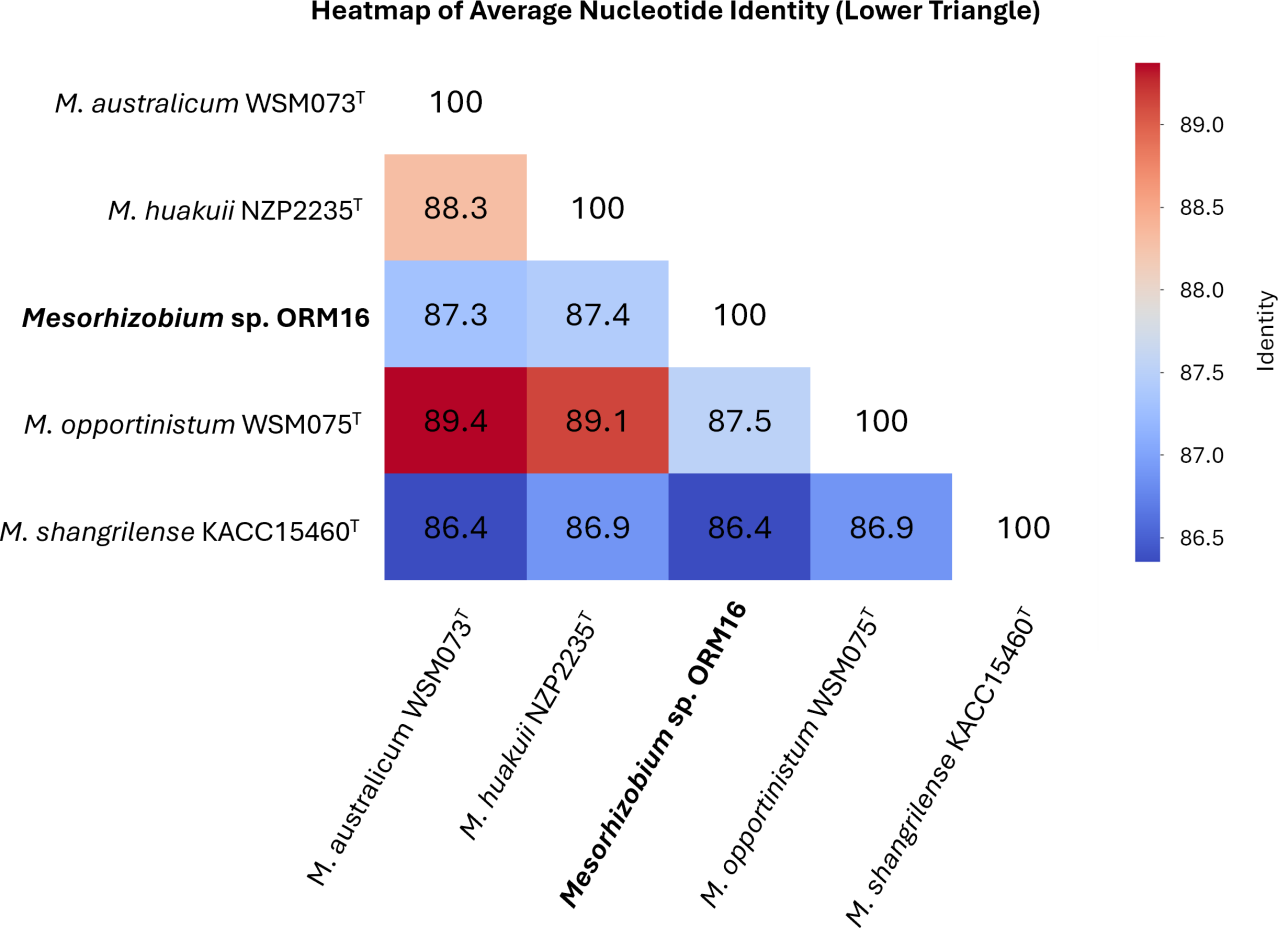
Genomic relatedness of *Mesorhizobium* sp. strain ORM16 to closest type strains based on ANI analysis

We performed Average Nucleotide Identity (ANI) analysis using the pyani command (v.0.3.0-alpha) (12). This computational approach allowed us to determine ANI percentages between our isolates and closely related reference strains while simultaneously constructing a phylogenetic tree based on these relationships. Sequencing analysis confirmed that the strain belongs to the genus *Mesorhizobium*, with the best match type strain being *Mesorhizobium opportunistum* WSM2075^T^, exhibiting an ANI of 87.23% (Fig. 1). Genome annotation using the NCBI PGAP pipeline identified 6,585 protein-coding sequences, 6 ribosomal RNA operons, 51 transfer RNAs, and 1 transfer-messenger RNA (tmRNA). The genes responsible for nodulation and nitrogen fixation were located on the chromosomal DNA.

Genomic distinctiveness makes ORM16 an ideal candidate for expanding our knowledge of *Mesorhizobium* diversity, taxonomic research, and exploring novel biotechnological applications in nitrogen fixation.

## Data Availability

Genomic sequences for the new *Mesorhizobium* sp. strain ORM16 have been submitted to GenBank (accession number: NZ_CP173356.1), BioProject PRJNA1178300, and BioSample SAMN44467667. Unprocessed sequencing reads are archived in the SRA database under accession SRR31173793.
